# A novel diagnostic strategy of differential diagnosis of tuberculous meningitis and non-tuberculous meningitis: a retrospective observational cohort study

**DOI:** 10.1128/spectrum.01898-25

**Published:** 2025-11-28

**Authors:** Qingwen Lin, Wenhua Fang, Kengna Fan, Weiqing Zhang, Xiaxia Qiu, Minjie Tang, Qi Wang, Huangcheng Shangguan, Qishui Ou, Xiaofeng Liu

**Affiliations:** 1 Department of Laboratory Medicine, Gene Diagnosis Research Center, The First Affiliated Hospital, Fujian Medical University 74551, Fuzhou, China; 2 Department of Laboratory Medicine, National Regional Medical Center, Binhai Campus of the First Affiliated Hospital, Fujian Medical University 74551, Fuzhou, China; 3 Fujian Key Laboratory of Laboratory Medicine, The First Affiliated Hospital, Fujian Medical University 74551, Fuzhou, China; 4 Fujian Clinical Research Center for Clinical Immunology Laboratory Test, The First Affiliated Hospital, Fujian Medical University 74551, Fuzhou, China; 5 Department of Neurosurgery, Neurosurgery Research Institute, The First Affiliated Hospital, Fujian Medical University 74551, Fuzhou, China; 6 Department of Neurosurgery, Binhai Branch of National Regional Medical Center, The First Affiliated Hospital, Fujian Medical University 74551, Fuzhou, China; 7 Department of Laboratory Medicine, Geriatric Hospital Affiliated with Wuhan University of Science and Technology 834316, Wuhan, China; 8 Department of Blood Transfusion, Geriatric Hospital Affiliated with Wuhan University of Science and Technology, Wuhan, China; Assistance Publique-Hopitaux de Paris Universite Paris Saclay, Clamart, France

**Keywords:** tuberculous meningitis, non-tuberculous meningitis, diagnosis, model

## Abstract

Tuberculous meningitis (TBM) leads to severe disability and mortality rates, making early diagnosis critical. However, it is challenging to distinguish it from other common forms of non-tuberculous meningitis (non-TBM), including bacterial meningitis, cryptococcal meningitis, and viral meningitis. This study aims to construct a diagnostic model between TBM and non-TBM. A total of 543 patients were enrolled, and 405 subjects remained and were subsequently divided into a training set and a validation set. Basic information, laboratory results, and imaging results of patients were collected, and R4.1.0 was used to construct and validate the diagnostic model. Subsequently, 30 patients were recruited as an independent validation cohort to verify the diagnostic efficacy of the model further. Finally, 10 patients with suspected TBM were prospectively observed, and the model was applied for diagnosis, with results compared to the final clinical diagnosis. The differential model of TBM and non-TBM was composed of the systemic symptoms of tuberculosis, altered consciousness, neurological deficits, meningeal irritation, cerebrospinal fluid protein, positive T-cell spot test for tuberculosis infection, and C-reactive protein. The areas under the receiver operating characteristic curve for the model in the training and validation sets were 0.872 (95% confidence interval [CI] = 0.833–0.913) and 0.844 (95% CI = 0.751–0.937), respectively. Furthermore, the validation cohort also shows good diagnostic performance with a sensitivity and specificity of 88.9% and 85.7%, respectively. Notably, 9 out of 10 patients had diagnoses consistent with model predictions. A novel diagnostic model was developed and validated using common clinical indicators and laboratory results to distinguish between TBM and non-TBM effectively.

**IMPORTANCE:**

Tuberculous meningitis is a serious disease. Currently, there is no effective way to perform early differential diagnosis, particularly in resource-limited settings. This article presents a new, simple method.

## INTRODUCTION

Tuberculous meningitis (TBM), a severe form of extrapulmonary tuberculosis, accounts for 5%–10% of all extrapulmonary cases. The combined mortality and disability rate can reach 50% (1). During the advanced stage of TBM, there may be a manifestation of altered mental status and focal neurological deficits. Complications such as cerebral ischemia, hydrocephalus, and cerebral infarction can lead to significant brain injury and neurological dysfunction (2, 3). These complications are primarily due to the difficulties in achieving early diagnosis and initiating appropriate treatment.

Differentiating TBM from bacterial meningitis (BM), viral meningitis (VM), and cryptococcal meningitis (CM) is particularly challenging in early stages due to non-specific clinical, imaging, and cerebrospinal fluid (CSF) findings. Although the pathogenic microorganisms remain the gold standard for diagnosing TBM, its sensitivity is notably inadequate and the process is often time-consuming, typically requiring 2–6 weeks for results (4, 5). Despite its high sensitivity and WHO endorsement, the utilization of GeneXpert MTB/RIF Ultra remains limited in recent years, primarily because of its high cost (6). Consequently, the differential diagnosis between TBM and non-tuberculous meningitis (non-TBM) remains a significant challenge, highlighting the urgent need for novel tools to facilitate the timely identification of TBM.

Recent studies have proposed new methods for early TBM diagnosis, including novel biomarkers such as lipids (7), proteins (8), and RNA (9). However, these methods are complex and expensive to operate. In terms of imaging, characteristic signs such as tuberculomas are rare, and more common signs like meningeal enhancement and vascular damage lack specificity (10). Moreover, the development of models that integrate multiple indicators has demonstrated significant value in disease diagnosis (11, 12). Meanwhile, nomograms, a visual representation of a model, have attracted much attention in the medical field (13). Although several studies have developed diagnostic nomograms for TBM (14–16), most have either omitted imaging findings or lacked thorough comparisons with common mimics like BM, VM, and CM. Thus, we expect to find a simple and practical new strategy for the differential diagnosis of TBM and non-TBM.

In this study, we retrospectively collected clinical information, common laboratory indicators, and imaging data from patients with meningitis in the First Affiliated Hospital of Fujian Medical University. We analyzed the characteristics of patients with TBM and those with non-TBM to identify relevant variables. Subsequently, we constructed a differential model to differentiate between TBM and non-TBM, aiming to provide a novel approach for the early diagnosis of TBM.

## MATERIALS AND METHODS

### Study subjects

This study retrospectively enrolled patients diagnosed with meningitis at the First Affiliated Hospital of Fujian Medical University between May 2018 and May 2023. We developed and validated this model following the protocol set out according to the TRIPOD statement.

**Fig 1:**
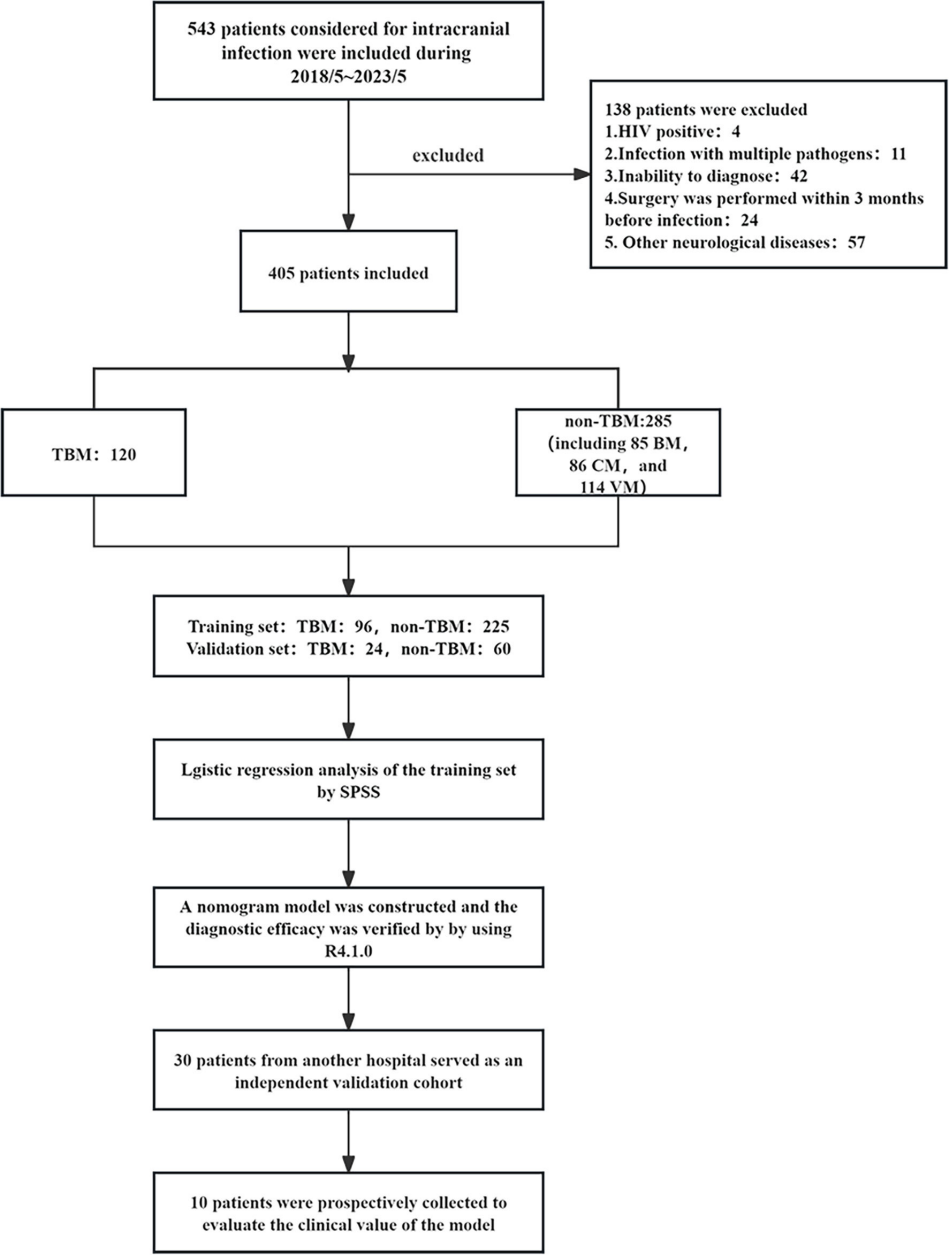
The flow chart of the present study. BM, bacterial meningitis; CM, cryptococcal meningitis; non-TBM, non-tuberculous meningitis; TBM, tuberculous meningitis; VM, viral meningitis.

We enrolled 543 cases of patients suspected of having intracranial infections. After careful selection, 405 subjects were included and divided into a training set and a validation set (Fig. 1). The inclusion and exclusion criteria were as follows.

The detailed diagnostic criteria for TBM, BM, CM, or VM included in this study were described in our previous study (17) as follows: (i) the diagnostic criteria for TBM were based on the 2010 international expert consensus on tuberculous meningitis; patients with definite and probable TBM were included in this study (18); (ii) CSF cryptococcus culture, ink-stained smear, and cryptococcal antigen positivity in meningitis patients were diagnosed as CM; (iii) the diagnosis of BM, on the one hand, was based on the positive results of at least one of the following tests: culture of pathogenic bacteria, PCR, or mNGS analysis from CSF, and on the other hand, it was based on the clinical symptoms, typically abnormal CSF findings (elevated white blood cell count, predominantly neutrophils, increased protein levels, decreased chloride levels, and reduced glucose levels, with glucose often falling below 2.2 mmol/L), and a good treatment effect of antibiotics; (iv) the VM was diagnosed based on positive pathogen tests from PCR or mNGS or the diagnosis was based on clinical judgment (normal or mildly abnormal CSF, good response to antiviral therapy, and exclusion of other causes of meningitis).

In addition, the exclusion criteria for this study were as follows: (i) HIV-positive patients, (ii) neurosurgery within 3 months, (iii) co-infection, (iv) combination or eventual diagnosis of other neurological diseases, and (v) inability to diagnose.

Subsequently, we collected 30 patients (9 TBM and 21 non-TBM) considered for meningitis from the Fujian Hospital of Huashan Hospital, Fudan University, between May 2023 and May 2024 as an independent validation cohort. Additionally, we prospectively collected 10 cases of suspected TBM from January 2024 to November 2024 for a case study. The aim is to further examine whether the constructed model can prospectively identify TBM patients in clinical applications.

### Data collection

Collect clinical information (age, gender, history, systemic symptoms of TB clinical performance, etc.). Among the clinical indicators, systemic symptoms of TB mainly include one or more of the following: weight loss, night sweats, prolonged low fever, and persistent cough for >2 weeks, imaging results (hydrocephalus, meningeal enhancement, cerebral infarction, tuberculoma, etc.), and laboratory indicators (CSF, blood common indicators, etc.) of patients upon admission from the electronic medical record system. The diagnosis of the enrolled patients was finally judged by two experienced clinicians based on the above diagnostic criteria, respectively, and grouped sequentially.

### Statistical analysis

This study used G*Power to estimate the sample size. The statistical power was assumed to be 95%, and the significance level was 0.05. All statistical tests were carried out on IBM SPSS 22.0 (IBM Corp.). Frequency and percentages were presented for categorical variables. The chi-square test or Fisher’s exact test was used for comparison. Continuous variables were reported as mean with SD or median with interquartile range, and the *t*-test or the Mann–Whitney *U* test was used to compare the intracranial infection group to the non-intracranial infection group. Missing data will be handled using multiple imputations. Further, variables with a *P* value of less than 0.05 in the univariate analysis were considered as candidates for inclusion. These candidate variables were entered into a multivariate logistic regression model. The final model was refined using a stepwise elimination approach that iteratively removes the least significant variables to achieve a parsimonious model with the best fit. Subsequently, the nomogram was constructed using R4.1.0 (General Public License) according to selected variables. Finally, the receiver operating characteristic (ROC) curve, calibration curve, and decision curve analysis (DCA) were conducted to evaluate the nomogram’s distinguishing efficacy and clinical usefulness in the training and validation set. A two-sided *P* < 0.05 was considered statistically significant.

## RESULT

### Clinical and demographic characteristics of the participants

The clinical characteristics of the TBM and non-TBM patients are shown in Table 1. In the training set, compared to the non-TBM group, the TBM group was older and had a higher proportion of patients with symptom duration between 10 and 30 days at admission (all *P* < 0.05). In terms of clinical manifestations, the TBM group exhibited significantly higher rates of systemic symptoms, altered consciousness, neurological deficits, and positive meningeal irritation compared to the non-TBM group (all *P* < 0.05). Conversely, the non-TBM group markedly increased the proportion of headaches and vomiting (all *P* < 0.05). Among the imaging findings, the positive rates of hydrocephalus, meningeal enhancement, tuberculoma, and extracranial tuberculosis in the TBM group were significantly higher than those in the non-TBM group (all *P* < 0.05). No significant differences were observed between the two groups regarding the remaining variables (all *P* > 0.05).

**TABLE 1:** Comparison of baseline information between TBM and non-TBM groups^*b*^*^,c^*

Information	TBM (*n* = 96)	Non-TBM				*P* value
		Total (*n* = 225)	VM (*n* = 91)	CM (*n* = 67)	BM (*n* = 67)	
Admission characteristics						
Male (*n* [%])	64 (66.7)	144 (64)	55 (60.4)	49 (73.1)	40 (59.7)	0.647
Age (*m* ± SD [yr])	50.84 ± 16.40	43.66 ± 19.11	36.01 ± 18.77	52.67 ± 14.43	45.03 ± 19.68	0.001^*a*^
History of tuberculosis contact (*n* [%])	7 (7.3)	6 (2.7)	1 (1.1)	4 (6.0)	1 (1.5)	0.054
Duration of symptoms (10–30 days) (*n* [%])	45 (46.9)	73 (32.4)	26 (28.6)	30 (44.8)	17 (25.4)	0.014^*a*^
ICP (*m* [IQR] [mmHg])	200 (146–288)	198 (127–270)	160 (111–222)	270 (149–330)	205 (148–260)	0.552
Clinical data (*n* [%])						
Systemic symptoms	22 (22.9)	9 (4.0)	4 (4.4)	3 (4.5)	2 (3.0)	<0.001^*a*^
Headache	74 (77.1)	196 (87.1)	77 (84.6)	59 (88.1)	60 (89.6)	0.024^*a*^
Vomiting	29 (30.2)	97 (43.1)	32 (35.2)	32 (47.8)	33 (49.3)	0.03^*a*^
Fever	75 (78.1)	165 (73.3)	77 (84.6)	29 (43.3)	59 (88.1)	0.365
Convulsions	6 (6.3)	19 (8.4)	10 (11.0)	3 (4.5)	6 (9.0)	0.502
Altered consciousness	50 (52.1)	71 (31.6)	19 (20.9)	22 (32.8)	30 (44.8)	0.001^*a*^
Neurological deficits	41 (42.7)	41 (18.2)	6 (6.6)	23 (34.3)	12 (17.9)	<0.001^*a*^
Meningeal irritation	80 (83.3)	158 (70.2)	65 (71.4)	40 (59.7)	53 (79.1)	0.014^*a*^
Imaging information (*n* [%])						
Hydrocephalus	18 (18.8)	12 (5.3)	0 (0)	7 (10.4)	5 (7.5)	<0.001^*a*^
Basal meningeal enhancement	36 (37.5)	56 (24.9)	18 (19.8)	24 (35.8)	14 (20.9)	0.022^*a*^
Tuberculoma	3 (3.1)	0 (0)	0 (0)	0 (0)	0 (0)	0.008^*a*^
Infarctions	9 (9.4)	9 (4)	1 (1.1)	5 (7.5)	3 (4.5)	0.055
Pre-contrast basal hyperdensity	1 (1.0)	1 (0.4)	0 (0)	1 (1.5)	0 (0)	0.534
Evidence of tuberculosis elsewhere	10 (10.4)	10 (4.5)	1 (1.1)	6 (9.0)	3 (4.5)	0.044^*a*^

^*a*^ *P *< 0.05.

^*b*^ BM, bacterial meningitis; CM, cryptococcal meningitis; ICP, intracranial pressure; IQR, interquartile range; *m*, median; non-TBM, non-tuberculous meningitis; SD, standard deviation; TBM, tuberculous meningitis; VM, viral meningitis.

^*c*^ History of tuberculosis contact denotes the history of recent (within past year) close contact with an individual with pulmonary tuberculosis or a positive TST or IGRA. Systemic symptoms included one or more of the following suggestive of tuberculosis: weight loss (or poor weight gain in children), night sweats, or persistent cough for more than 2 weeks.

### Laboratory indicators of the included participants

Furthermore, we compared the common laboratory indicators between the two groups. As shown in Table 2, compared to the non-TBM group, the TBM group exhibited significantly lower levels of white blood cell count, neutrophil count, lymphocyte count, monocyte count, C-reactive protein (CRP), procalcitonin, sodium, chloride, calcium, uric acid, activated partial thromboplastin time, and fibrinogen (all *P* < 0.05). However, the TBM group showed significantly higher rates of TB antibodies, positive T-cell spot test for tuberculosis infection (T-SPOT) results, and levels of D-dimer compared to the non-TBM group (all *P* < 0.05). Additionally, CSF chloride and CSF glucose/blood glucose ratio were markedly decreased in the TBM group (all *P* < 0.05), while CSF glucose, CSF protein, and CSF lactate were markedly decreased in the non-TBM group (all *P* < 0.05). There were no significant differences observed between the two groups in terms of the remaining variables (all *P* > 0.05).

**TABLE 2:** Comparison of laboratory indexes between TBM group and non-TBM group^*b*^

Laboratory investigations	TBM (*n* = 96)	Non-TBM				*P* value
		Total (*n* = 225)	VM (*n* = 91)	CM (*n* = 67)	BM (*n* = 67)	
Blood (*m* [IQR])						
Leukocyte (10^9^/L)	7.89 (5.83–9.84)	9.51 (7.05–12.97)	8.21 (6.40–10.35)	9.23 (6.63–12.05)	12.97 (9.69–18.44)	0.001^*a*^
NE (%)	78.4 (67.1–84.9)	80.2 (68.5–86.7)	71.9 (59.5–82.9)	80.5 (76.0–85.4)	84.9 (79.4–90.1)	0.508
NE (10^9^/L)	5.71 (3.94–8.15)	7.31 (5.00–10.58)	5.92 (3.64–8.17)	7.71 (5.26–9.94)	9.69 (6.94–15.77)	0.002^*a*^
Lymphocytes (%)	12.0 (8.1–19.6)	11.6 (7.2–20.7)	17.2 (11.1–27.9)	10.3 (8.0–14.4)	8.6 (4.6–12.3)	0.662
Lymphocytes (10^9^/L)	0.97 (0.64–1.45)	1.16 (0.75–1.71)	1.51 (0.98–1.91)	0.94 (0.64–1.28)	1.06 (0.65–1.68)	0.018^*a*^
Monocytes (%)	5.8 (4.6–7.4)	5.5 (3.9–6.9)	5.8 (4.5–7.25)	5.7 (4.1–7.2)	4.4 (3.6–6.5)	0.118
Monocytes (10^9^/L)	0.43 (0.35–0.59)	0.49 (0.36–0.67)	0.45 (0.35–0.57)	0.45 (0.38–0.63)	0.57 (0.37–0.93)	0.049^*a*^
RBC (10^12^/L)	4.39 (4.01–4.71)	4.44 (3.99–4.78)	4.52 (4.21–4.87)	4.43 (4.00–4.84)	4.24 (3.83–4.71)	0.629
Hemoglobin (g/L)	130.27 ± 17.12	131.17 ± 18.32	136.20 ± 16.29	134.25 ± 19.16	126.20 ± 19.37	0.691
Hematocrit (L/L)	0.378 ± 0.047	0.381 ± 0.050	0.387 ± 0.045	0.387 ± 0.050	0.368 ± 0.054	0.532
RDW (%)	13.2 (12.3–14.1)	13.2 (12.4–14.0)	12.9 (12.3–13.8)	13.8 (12.8–14.5)	13.3 (12.3–14.1)	0.72
HDW (g/L)	24.6 (23.1–26.6)	25.2 (23.5–27.6)	24.9 (23.5–27.0)	25.1 (23.3–27.8)	25.6 (23.5–27.9)	0.088
Platelet (10^9^/L)	230.0 (186–287)	227.0 (177–305)	233.0 (182–375)	222.5 (176–302)	212.0 (163–344)	0.851
MPV (fL)	8.2 (7.5–9.1)	8.4 (7.6–9.2)	8.3 (7.5–9.4)	8.4 (7.6–9.0)	8.4 (7.5–9.1)	0.629
PDW (%)	50.2 (45.2–54.4)	51.0 (45.9–56.5)	51.2 (45.9–57.2)	50.8 (45.7–56.3)	51.8 (46.5–56.7)	0.259
Plateletcrit (%)	0.19 (0.15–0.24)	0.19 (0.15–0.24)	0.20 (0.17–0.24)	0.19 (0.14–0.24)	0.19 (0.14–0.24)	0.461
MPC (g/L)	263.0 (241–277)	260.0 (242–275)	263.0 (241–280)	254.0 (238–273)	259.0 (248–270)	0.654
TB-Ab positive (*n* [%])	12 (12.5)	8 (3.6)	3 (3.3)	2 (3.0)	3 (4.5)	0.002^*a*^
T-SPOT positivity (*n* [%])	45 (46.9)	28 (12.4)	9 (9.9)	9 (13.4)	10 (14.9)	<0.001^*a*^
CRP (mg/L)	6.24 (5.00–15.46)	12.55 (5.00–37.80)	5.02 (5.00–18.9)	7.55 (5.00–16.15)	80.0 (21.11–90.0)	0.004^*a*^
PCT (ng/mL)	0.05 (0.05–0.06)	0.05 (0.05–0.17)	0.05 (0.05–0.06)	0.05 (0.04–0.06)	0.30 (0.05–5.98)	0.011^*a*^
Glucose (mmol/L)	6.30 (5.43–7.78)	6.21 (5.27–7.71)	5.85 (5.01–6.87)	6.28 (5.46–8.21)	7.14 (5.71–8.43)	0.663
Potassium (mmol/L)	3.96 (3.65–4.36)	3.92 (3.59–4.22)	3.99 (3.67–4.33)	3.98 (3.52–4.22)	3.79 (3.51–4.14)	0.397
Sodium (mmol/L)	132.6 (127.7–137.5)	137.1 (133.6–140.0)	137.6 (135.3–140.1)	135.6 (133.0–139.0)	137.6 (133.8–140.5)	<0.001^*a*^
Chloride (mmol/L)	95 (90–98)	99 (95–102)	100 (96–102)	98 (92–100)	98 (96–102)	<0.001^*a*^
Calcium (mmol/L)	2.19 (2.11–2.28)	2.25 (2.15–2.36)	2.28 (2.17–2.38)	2.23 (2.13–2.36)	2.25 (2.14–2.34)	0.002^*a*^
Urea (mmol/L)	4.54 (3.36–5.68)	4.48 (3.55–6.08)	4.21 (3.34–5.15)	4.80 (3.58–6.66)	4.96 (3.68–6.08)	0.278
Creatinine (μmol/L)	56.4 (46.9–70.6)	60.8 (49.6–73.1)	63.5 (48.4–72.5)	60.2 (48.7–72.1)	60.8 (52.1–78.2)	0.106
Uric acid (μmol/L)	180.7 (119.0–268.8)	231.1 (154.1–318.4)	232.3 (150.3–323.3)	203.0 (143.7–322.5)	250.2 (184.65–311.2)	0.001^*a*^
D-dimer (ug/L)	0.84 (0.48–1.82)	0.62 (0.34–1.47)	0.46 (0.27–1.14)	0.44 (0.31–1.07)	1.30 (0.60–2.95)	0.037^*a*^
PT (s)	11.8 (11.4–12.5)	12.1 (11.4–12.9)	12.2 (11.5–12.6)	11.6 (11.1–12.6)	12.7 (11.9–13.7)	0.068
INR	1.03 (0.99–1.09)	1.04 (0.98–1.12)	1.04 (1.00–1.09)	1.01 (0.96–1.08)	1.11 (1.01–1.17)	0.564
APTT (s)	25.5 (23.1–28.1)	27.3 (23.7–30.5)	27.2 (23.7–29.4)	25.3 (22.4–29.6)	28.9 (25.0–34.3)	0.019^*a*^
Fg (g/L)	3.13 (2.6–4.05)	3.67 (2.76–4.92)	3.03 (2.35–4.31)	3.54 (2.82–4.31)	5.09 (3.96–6.95)	0.004^*a*^
TT (s)	15.8 (15.0–16.8)	15.6 (15.0–16.6)	15.9 (15.2–16.8)	15.8 (15.2–16.7)	15.2 (14.4–15.7)	0.414
Albumin (g/L)	39.03 ± 4.82	39.32 ± 4.74	39.70 ± 4.35	39.03 ± 5.11	39.10 ± 4.91	0.253
Globulin (g/L)	25.0 (22.3–28.9)	25.9 (23.6–29.2)	25.1 (23.0–27.8)	25.6 (23.3–28.7)	27.4 (25.1–31.8)	0.172
NSE (ng/mL)	11.97 (8.89–17.51)	10.96 (7.34–14.18)	12.07 (9.11–16.54)	12.05 (8.36–14.89)	9.76 (7.04–12.89)	0.056
CSF (*m* [IQR])						
Leukocyte (10^6^/L）	195 (96.5–378)	163 (55–439)	76 (37–177)	140 (57–272)	676 (201–3,020)	0.246
Chloride (mmol/L)	109 (105–114)	116 (112–120)	118 (115–120)	113 (109–116)	117 (113–121)	<0.001^*a*^
Glucose (mmol/L)	2.55 (1.99–3.21)	2.08 (1.66–2.44)	2.90 (2.48–3.29)	2.08 (1.13–2.59)	2.25 (1.17–3.29)	<0.001^*a*^
Protein (g/L)	2.00 (1.34–2.92)	1.02 (0.52–2.29)	0.66 (0.38–0.98)	1.29 (0.68–3.0)	2.18 (1.03–3.0)	<0.001^*a*^
Lactate (mmol/L)	3.80 (2.72–5.5)	2.72 (1.8–5.1)	1.8 (1.5–2.2)	4.15 (3.22–5.68)	5.53 (2.61–9.98)	0.001^*a*^
RBC (10^9^/L)	0.1 (0.1–0.23)	0.1 (0–0.3)	0.1 (0–0.2)	0.1 (0.1–0.3)	0.3 (0.1–0.7)	0.519
GluR (%)	0.33 (0.23–0.42)	0.43 (0.27–0.53)	0.49 (0.42–0.58)	0.27 (0.18–0.45)	0.36 (0.18–0.49)	0.001^*a*^

^*a*^ *P *< 0.05.

^*b*^ APTT, activated partial thromboplastin time; CRP, C-reactive protein; CSF, cerebrospinal fluid; Fg, fibrinogen; GluR, the CSF/blood glucose ratio; HDW, hemoglobin distribution width; INR, international normalized ratio; MPC, mean platelet content concentration; MPV, mean platelet volume; NE, neutrophil; NSE, neuron-specific enolase; PCT, procalcitonin; PDW, platelet distribution width; PT, prothrombin time; RBC, red blood cell; RDW, RBC distribution width; TB-Ab, TB antibody; TT, thrombin time.

### Development of the differential diagnosis model

**Fig 2:**
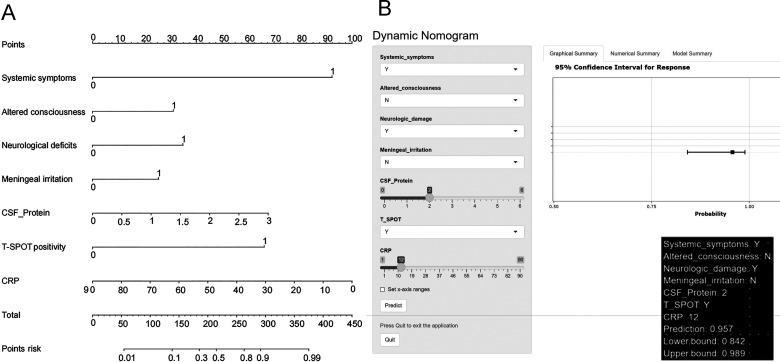
Nomogram for identifying TBM and non-TBM. A vertical line was drawn from the axis of each variable value to the first axis marked “points,” and the corresponding fraction was observed. The patient’s total score was summarized and matched to the last axis, and the differential diagnosis of the patient was made by the final probability. Defining categorical variables: 0 = no, 1 = yes. (**A**) Diagnostic nomogram for identifying TBM from non-TBM. (**B**) The web-based dynamic nomogram (https://nomogram-pnici.shinyapps.io/DynNomapp_2/). CRP, C-reactive protein; CSF, cerebrospinal fluid; T-SPOT, T-cell spot test for tuberculosis infection.

Following the univariate analysis, the variables that presented statistical differences were subjected to multivariate logistic regression analysis. The final predictive variables were selected through a stepwise algorithm, yielding a parsimonious model with seven independent predictors. As detailed in Table 3, these predictors were the system symptoms of tuberculosis, altered consciousness, neurological deficits, meningeal irritation, CSF protein levels, positive T-SPOT results, and serum CRP (all *P* < 0.05). Notably, conventional CSF parameters (glucose, chloride, and leukocyte count) were excluded during stepwise selection due to lack of independent significance (all *P* > 0.05) after adjustment, suggesting their predictive value was captured by stronger predictors. The variables were input into R4.1.0 software, and the rms package was used to construct a nomogram. The nomogram displays the scores of the seven variables and the corresponding likelihood of diagnosing TBM based on the total score obtained by summing the scores of each variable. A higher total score indicates a higher likelihood of diagnosing TBM (Fig. 2A). Additionally, a dynamic nomogram that can be accessed online has been created (https://nomogram-pnici.shinyapps.io/DynNomapp_2/, Fig. 2B).

**TABLE 3:** Variables were screened by multiple regression^*a*^

	*B*	S.E.	Wald	df	*P*	OR	95% CI
Systemic symptoms	3.144	0.638	24.306	1	<0.001	23.204	6.648–80.989
Altered consciousness	0.859	0.337	6.509	1	0.011	2.361	1.22–4.568
Neurological deficits	0.899	0.355	6.421	1	0.011	2.456	1.226–4.921
Meningeal irritation	0.821	0.406	4.082	1	0.043	2.273	1.025–5.042
CSF protein	0.694	0.189	13.556	1	<0.001	2.002	1.384–2.897
T-SPOT positivity	1.85	0.36	26.365	1	<0.001	6.358	3.138–12.882
CRP	−0.034	0.007	22.075	1	<0.001	0.966	0.953–0.98
Intercept	−3.13	0.733	18.25	1	<0.001	0.044	

^*a*^ CI, confidence interval; CRP, C-reactive protein; CSF, cerebrospinal fluid; OR, odds ratio.

### Validation of the diagnostic model

**Fig 3:**
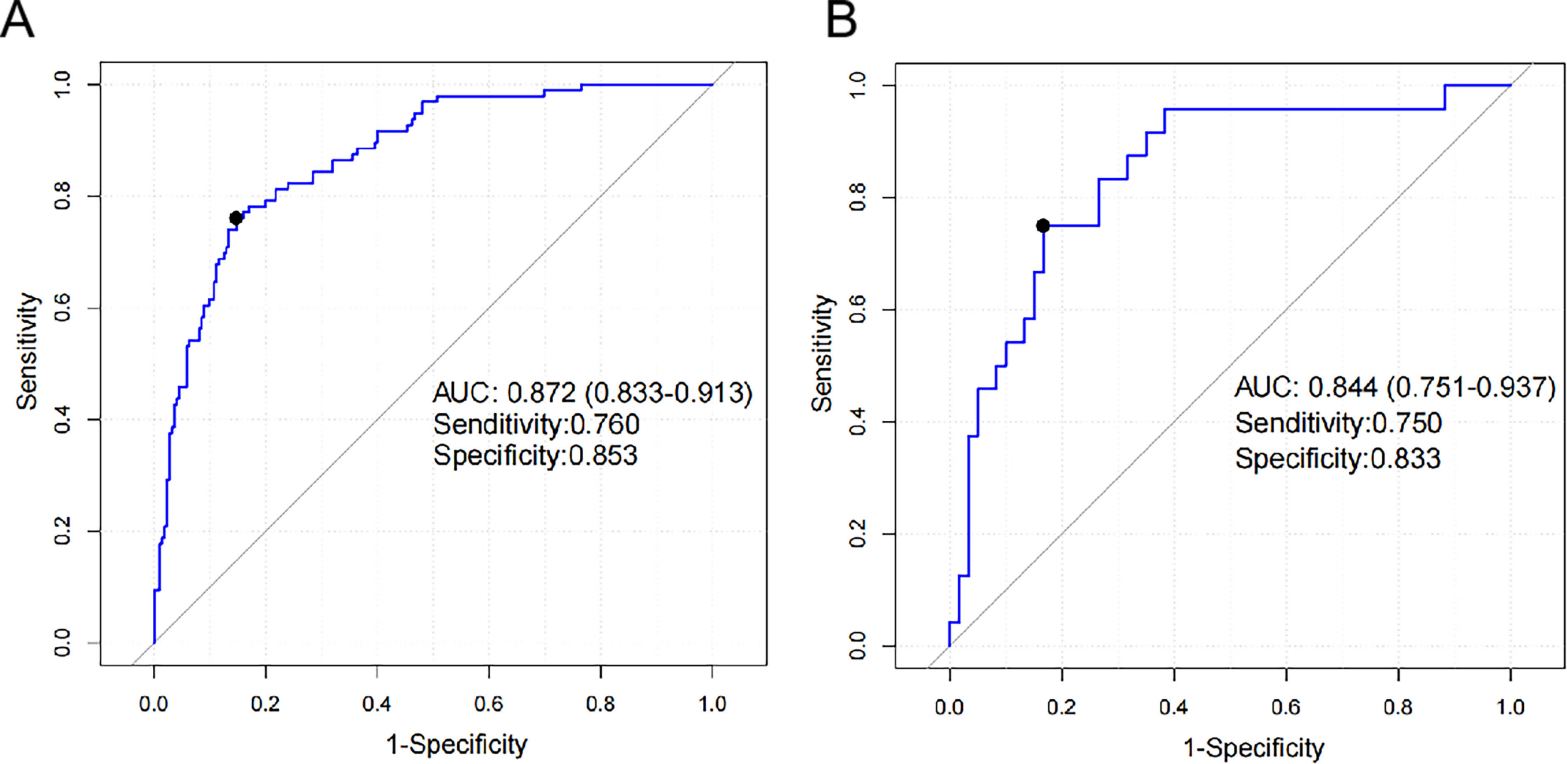
Distinguishing efficiency of the model between TBM and non-TBM. (**A**) ROC curve of the training set; the area under the receiver operating characteristic curve (AUC) was 0.872; the sensitivity was 76.0%; and the specificity was 85.3%. (**B**) ROC curve of the validation set; the AUC was 0.844; the sensitivity was 75.0%; and the specificity was 83.3%

Finally, we evaluated the diagnostic performance of the nomogram on both the training and validation sets. The nomogram exhibited favorable diagnostic ability in both the training and validation sets. The ROC curve (Fig. 3A and B) showed that the areas under the receiver operating characteristic curve were 0.872 (95% confidence interval [CI] = 0.833–0.913) and 0.844 (95% CI = 0.751–0.937). The sensitivity was 76.0% and 75.0%, and the specificity was 85.3% and 83.3% in the training and validation sets, respectively.

**Fig 4:**
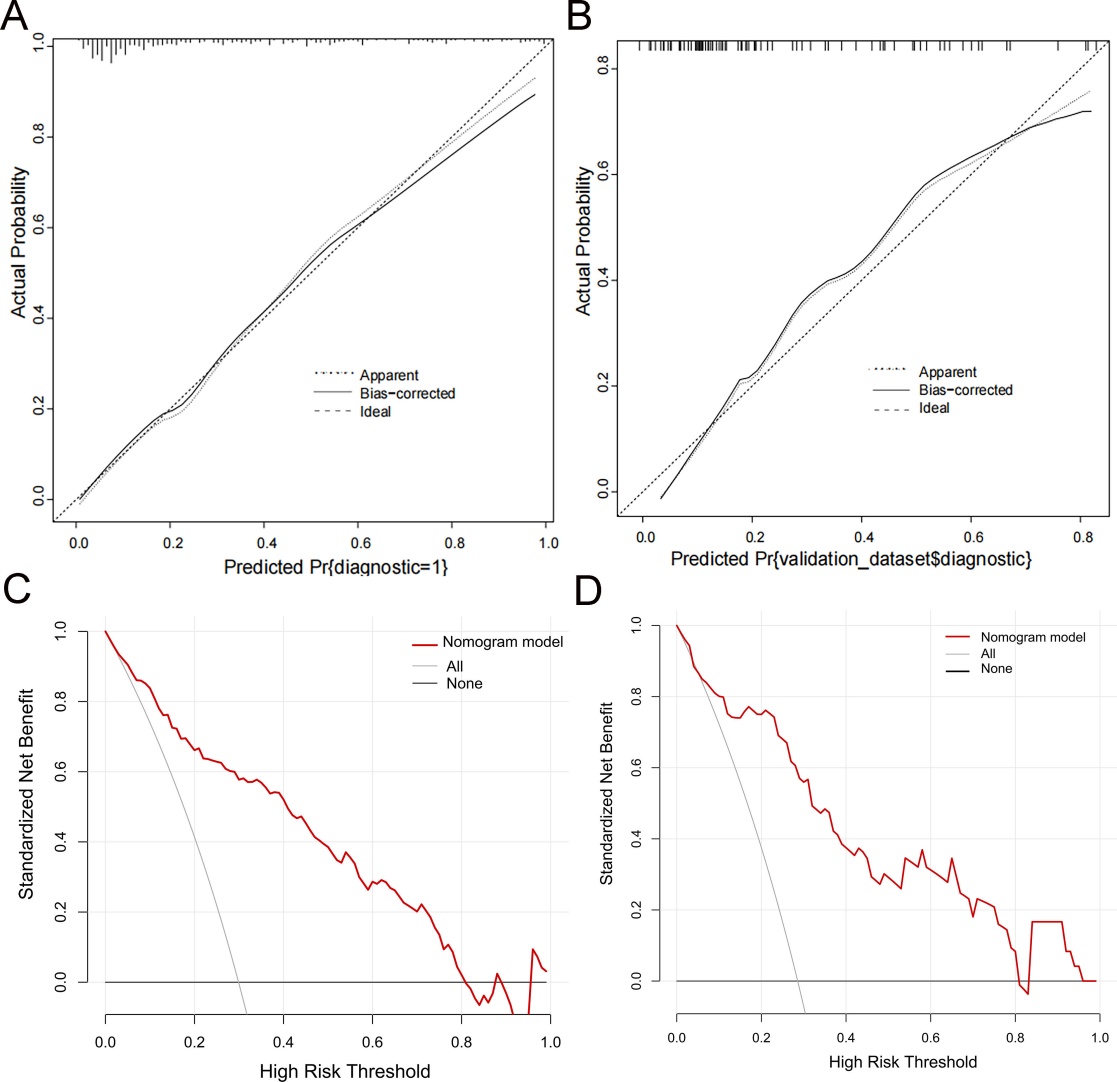
Calibration and decision curve analysis (DCA) of the model. (**A and B**) The *y*-axis represents the observed TBM, and the *x*-axis represents the probability of TBM. The dotted line represents the apparent curve. The solid line is calculated by bootstrapping (resample: 1,000) and represents the discrimination for TBM using a nomogram. The diagonal dashed line represents the ideal line showing the diagnostic probability is consistent with the observed probability. (**A**) Training set’s calibration curve. (**B**) Validation set’s calibration curve. (**C and D**) The *y*-axis represents the net benefit, and the *x*-axis indicates the threshold probability. The solid red line shows the net benefit of relying on the model to diagnose and treat the patient. The gray line represents the gain when all patients were assumed to be treated as TBM. The black line represents the gain when all patients were assumed to be treated as non-TBM. (**C**) Training set’s DCA curve. (**D**) Validation set’s DCA curve.

Bootstrap calibration with 1,000 resamples showed that the predicted probability of TBM diagnosis closely aligned with the observed outcome, as indicated by the proximity of the calibration curve to the ideal line (Fig. 4A and B). Additionally, DCA curves were used to assess the clinical application values of the nomogram model of TBM (Fig. 4C and D). In the validation set, when the threshold probability was between 0.1 and 0.8, using the nomogram to diagnose TBM presented a greater net benefit than using the treat-all-patients scheme or the treat-none scheme (Fig. 4D).

### External validation of the model and prospective case studies

To evaluate the differential diagnostic efficacy of the model for TBM versus non-TBM, we included an independent validation cohort from the Fujian Hospital of Huashan Hospital, Fudan University. The validation cohort consisted of 9 cases in the TBM group and 21 cases in the non-TBM group, and their clinical characteristics and demographic information are shown in Table 4. A validation cohort analysis showed that the sensitivity and specificity of the model developed in this study for the differential diagnosis of TBM and non-TBM were 88.9% and 85.7%, respectively, as shown in Table 5. In addition, we prospectively collected 10 patients with suspected TBM for case follow-up. The aim was to compare the compliance of the prospective diagnostic results obtained using our established model with the final clinical diagnosis in order to assess the clinical utility of the model. As shown in Table 6, five patients were diagnosed with TBM; four patients were diagnosed with non-TBM; and one patient was difficult to classify but tended to have TBM by early judgment of the model. Ultimately, four cases were diagnosed as non-TBM and six as TBM, which is highly consistent with the results of the model. This suggests that the model has good clinical application value for diagnosing TBM.

**TABLE 4:** Basic information of independent validation cohort^*b*^^,^^*c*^

Information	TBM (*n* = 9)	Non-TBM (*n* = 21)	*P* value
		BM 6, CM 6, and VM 9	
Admission characteristics			
Male (*n* [%])	6 (66.7)	16 (76.2)	0.298
Age (*m* ± SD [yr])	52.67 ± 16.76	52.33 ± 13.19	0.786
Clinical data (*n* [%])			
Systemic symptoms	2 (22.2)	0 (0)	0.025^*a*^
Headache	6 (66.7)	15 (71.4)	0.794
Vomiting	2 (22.2)	7 (33.3)	0.543
Fever	6 (66.7)	10 (47.6)	0.338
Convulsions	0 (0)	0 (0)	N/A
Altered consciousness	7 (77.8)	7 (33.3)	0.025^*a*^
Neurological deficits	4 (44.4)	6 (28.6)	0.398
Meningeal irritation	9 (100.0)	11 (52.4)	0.011^*a*^
Imaging information (*n* [%])			
Hydrocephalus	2 (22.2)	0 (0)	0.025^*a*^
Basal meningeal enhancement	6 (66.7)	6 (28.6)	0.051
Tuberculoma	0 (0)	0 (0)	N/A
Infarctions	4 (44.4)	1 (4.8)	0.008
Pre-contrast basal hyperdensity	2 (22.2)	0 (0)	0.025^*a*^
Evidence of tuberculosis elsewhere	3 (33.3)	0 (0)	0.005^*a*^
CSF (*m* [IQR])			
Leukocyte (10^6^/L）	251 (137–429)	131 (82–281)	0.118
Chloride (mmol/L)	113 (109–119)	119 (113–122)	0.113
Glucose (mmol/L)	1.87 (1.20–2.94)	2.88 (2.03–3.02)	0.248
Protein (g/L)	1.81 (1.25–2.5)	1.13 (0.65–1.83)	0.046^*a*^
Lactate (mmol/L)	5.98 (4.82–10.56)	3.0 (1.93–5.17)	0.025^*a*^
GluR (%)	0.24 (0.15–0.43)	0.39 (0.30–0.57)	0.05^*a*^
Blood (*m* [IQR])			
Leukocyte (10^9^/L)	7.5 (5.9–9.4)	7.7 (6.1–9.9)	0.594
NE (%)	80.3 (76.6–88.1)	72.0 (60.2–86.7)	0.150
TB-Ab positive (*n* [%])	0 (0)	0 (0)	N/A
T-SPOT positivity (*n* [%])	8.0 (88.9)	4.0 (19.0)	<0.001^*a*^
CRP (mg/L)	2.3 (1.1–46.6)	3.4 (1.25–23.5 )	0.856

^*a*^ *P *< 0.05.

^*b*^ BM, bacterial meningitis; CM, cryptococcal meningitis; CSF, cerebrospinal fluid; GluR, the CSF/blood glucose ratio; *m*, median; NE, neutrophil; non-TBM, non-tuberculous meningitis; SD, standard deviation; TBM, tuberculous meningitis; VM, viral meningitis; N/A, that a comparison was not applicable because the positive rate was 0% for this item in both groups, owing to the small sample size in the independent validation cohort.

^*c*^ Systemic symptoms are those suggestive of tuberculosis (one or more of the following): weight loss (or poor weight gain in children), night sweats, or persistent cough for more than 2 weeks.

**TABLE 5:** Diagnostic efficacy of the model in a validated cohort^*a*^^,*b*^

Model diagnosis	Diagnostic criteria		Total
	TBM	Non-TBM	
TBM	8	3	11
Non-TBM	1	18	19
Total	9	21	

^*a*^ Non-TBM, non-tuberculous meningitis; TBM, tuberculous meningitis.

^*b*^ The diagnostic sensitivity, specificity, positive predictive value, and negative predictive value were 88.9%, 85.7%, 0.727, and 0.947, respectively.

**TABLE 6:** The performance of the model was tested on 10 patients^*a*^

Patients	Case 1	Case 2	Case 3	Case 4	Case 5	Case 6	Case 7	Case 8	Case 9	Case 10
Systemic symptoms	Yes	No	No	No	No	No	Yes	Yes	No	Yes
Altered consciousness	No	Yes	Yes	No	Yes	Yes	No	No	No	No
Neurological deficits	Yes	No	No	No	No	Yes	No	No	Yes	No
Meningeal irritation	Yes	Yes	Yes	Yes	Yes	Yes	Yes	Yes	Yes	Yes
CSF protein	0.79	1.77	1.19	1.14	3.0	2.51	2.29	3.0	3.0	0.7
T-SPOT positivity	No	Yes	No	Yes	No	Yes	Yes	No	No	No
CRP	10	5	7	7.14	58	3	5	2.5	8	90
Model prediction probability	0.78	0.77	0.25	0.46	0.17	0.94	0.96	0.88	0.53	0.09
Diagnosis	TBM	TBM	Non-TBM	Non-TBM	Non-TBM	TBM	TBM	TBM	TBM	Non-TBM

^*a*^ CRP, C-reactive protein; CSF, cerebrospinal fluid; non-TBM, non-tuberculous meningitis; TBM, tuberculous meningitis.

## DISCUSSION

TBM is the most severe form of extrapulmonary tuberculosis (19). Studies have shown that only 47% of TBM patients recover, highlighting the crucial role of early diagnosis in improving outcomes (20). Due to the atypical clinical presentation of TBM and the difficulties in pathogen culture, it is challenging to differentiate TBM from BM, VM, and CM. In this study, we developed and validated a novel scoring model by comparing a total of 64 factors, including clinical information, laboratory indicators, and imaging findings, between the TBM group and the non-TBM group, which provided a more comprehensive presentation of the differences between TBM and non-TBM patients. Ultimately, the model incorporated systemic symptoms of tuberculosis, altered consciousness, neurological deficits, meningeal irritation, CSF protein levels, T-SPOT results, and CRP. Our findings suggest that the model shows high diagnostic efficacy for TBM and holds promise as a new method for distinguishing between clinical TBM and common non-TBM cases.

The model constructed in this study includes the systemic symptoms of tuberculosis, altered consciousness, neurological impairment, and meningeal irritation, which are the relatively specific clinical manifestations of tuberculosis. However, systemic symptoms of tuberculosis are not in all patients; only 22.9% of patients in this study developed systemic symptoms, which is similar to the results of the spinal tuberculosis patients studied by Li et al. (21). Furthermore, CSF circulation disorders caused by TBM, subsequent hydrocephalus, and cerebral infarction may all result in altered consciousness (22, 23). Nerve damage is often caused by the accumulation of inflammatory exudates near the cranial nerves in TBM patients. Moreover, tuberculosis is highly disseminated and can spread downward to affect the spinal cord, resulting in peripheral nerve dysfunction. Meningeal irritation signs are typical manifestations of meningitis because of severe inflammation leading to meningeal irritation. This study found a higher proportion of meningeal irritation signs in the TBM group, possibly due to the more severe condition and intense inflammatory response in TBM patients.

In addition to clinical features, the model also included three laboratory indicators: T-SPOT, CRP, and CSF protein. T-SPOT detects effector T cells activated by *Mycobacterium tuberculosis* and its specific antigens to help determine if a patient is infected with tuberculosis. The test results are not affected by BCG vaccination. Previous studies have shown that T-SPOT has diagnostic value for various forms of extrapulmonary tuberculosis, including tuberculous meningitis (16, 24, 25). However, it is also crucial to acknowledge an important limitation of the T-SPOT as a diagnostic tool for TBM. T-SPOT exhibits high sensitivity, but its specificity can be compromised in high TB-burden settings (26, 27). This is because the test detects immune sensitization to *M. tuberculosis* but can neither differentiate between latent tuberculosis infection and active disease nor localize the infection to the central nervous system. This inherent characteristic of T-SPOT likely contributes to the observed misclassifications in our study and is a key reason why it must be interpreted as part of a multi-parameter algorithm rather than as a stand-alone diagnostic test for TBM. The strength of our model lies in its ability to integrate the probabilistic information from T-SPOT with other clinical and cerebrospinal fluid indicators to achieve a more robust overall diagnostic accuracy than any single marker could provide.

As an acute-phase protein, CRP rises in the body during infections and stress, especially in bacterial infections. Studies have shown that CRP rises significantly during bacterial infections (28, 29), aiding in distinguishing TBM from BM, which could be why CRP in our paper was higher in the non-TBM group than in the TBM group. Additionally, our study displayed that CSF protein levels in the TBM group are significantly higher than in the non-TBM group, especially in the VM and CM groups. This might be due to the predominant inflammatory exudation in TBM, leading to higher protein levels. The elevated protein levels are related to increased blood–brain barrier permeability. Typically, the more severe the inflammation, the more extensive the blood–brain barrier damage, consistent with the findings of Wang et al. (30) and Manyelo et al. (31). Further analysis across non-TBM subtypes confirmed the model’s broad utility. It showed strong performance in comparisons with bacterial and viral meningitis, while its discrimination from CM was more nuanced, consistent with the recognized diagnostic difficulty in this scenario (32, 33). This finding reinforces the model’s role not only as a stand-alone test but also as a robust initial aid that can effectively triage cases.

Finally, we used another independent validation cohort to further assess the diagnostic efficacy of the model for TBM. The results again validated the value of the model in differentiating between TBM and non-TBM. In addition, we prospectively collected information on 10 cases, classified them using the model, and tracked the patients’ final diagnoses. By comparing the two, we further evaluated the clinical utility of the model, and the results indicated a high concordance between the classification results of the model and the clinical final diagnoses, which implies that the model could aid in early differential diagnosis in clinical practice. It is necessary to continue expanding the number of patients in the prospective cohort and to continuously monitor the actual value of the model in clinical diagnosis, allowing the model to improve in line with changes in the clinical characteristics and epidemiology of TBM, thereby better assisting clinicians in their diagnosis and treatment. However, the broader application of this model, particularly in high-risk populations, remains to be fully explored. Of course, its broader application warrants further investigation. Particularly, given the heightened susceptibility and often atypical presentation of tuberculous meningitis in children, who represent a high-risk population with devastating outcomes, there is a pressing need to evaluate the performance and utility of this diagnostic algorithm in pediatric patients. Future studies focused on validating and potentially adapting our model for children will be crucial to ensure it can inform clinical decisions and improve prognosis across all age groups.

The early diagnosis of TBM remains a significant clinical challenge, prompting numerous studies to explore various strategies for achieving timely identification of this condition. While new biomarkers such as RNA (34, 35) and proteomics (8) have been proposed, their validation is still pending, and they often come with high costs. In contrast, the data required for our model are typically readily available and easily accessible, enhancing its practical applicability. Although several models beneficial for diagnosing TBM have been studied, they also have some limitations. For example, some studies have utilized a combination of tuberculostearic acid with clinical manifestations and laboratory tests for diagnosing TBM (15). However, tuberculostearic acid is not widely implemented in medical institutions. Other research has constructed models using clinical information and laboratory indicators to distinguish between TBM, VM, and BM (16). In contrast, our study incorporates a more extensive data set, providing a more comprehensive overview and encompassing a wider spectrum of diseases, including the common types of community-acquired meningitis: TBM, BM, VM, and CM.

However, this study has some limitations. First, the model is based on retrospective data and relies mainly on medical records, which may lead to underestimation of some clinical features of patients, such as systemic symptoms of tuberculosis, potentially causing inaccurate results. Second, some data were unavailable; for example, CSF cytology classification was not included in this study due to excessive missing data, limiting the collection of laboratory indicators. Third, most of the included BM patients in this study had high CSF white blood cell counts, indicating the need for further collection of atypical BM cases to validate the model’s performance. Most importantly, the external validation cohort, while independent, was relatively small and sourced from a single center. This limited sample size reduces the precision of our performance estimates and may limit the generalizability of our findings. Therefore, while our results are promising, they require confirmation in larger, multi-center, and geographically diverse cohorts. Future studies should therefore focus on validation in large-scale, prospective, multi-center cohorts. Furthermore, integrating such a validated model into electronic health records could potentially support early clinical diagnosis and intervention.

### Conclusion

This study developed a diagnostic model and a web-based calculator for the differential diagnosis of TBM based on seven variables, including clinical signs and laboratory indicators at admission. The model demonstrates good diagnostic performance, aiding in the early identification of TBM, which improves patient prognosis and optimizes the use of medical resources.

## Data Availability

Datasets of this work are available from the corresponding authors as required.
